# Experimental Evaluation of Machine Learning Methods for Robust Received Signal Strength-Based Visible Light Positioning

**DOI:** 10.3390/s20216109

**Published:** 2020-10-27

**Authors:** Willem Raes, Nicolas Knudde, Jorik De Bruycker, Tom Dhaene, Nobby Stevens

**Affiliations:** 1 ESAT-TELEMIC, KU Leuven, 9000 Ghent, Belgium; jorik.debruycker@kuleuven.be (J.D.B.); nobby.stevens@kuleuven.be (N.S.); 2 IDLab-Imec, UGent, 9000 Ghent, Belgium; nicolas.knudde@ugent.be (N.K.); tom.dhaene@ugent.be (T.D.)

**Keywords:** Visible Light Positioning, robust, machine learning, multi layer perceptron, gaussian processes

## Abstract

In this work, the use of Machine Learning methods for robust Received Signal Strength (RSS)-based Visible Light Positioning (VLP) is experimentally evaluated. The performance of Multilayer Perceptron (MLP) models and Gaussian processes (GP) is investigated when using relative RSS input features. The experimental set-up for the RSS-based VLP technology uses light-emitting diodes (LEDs) transmitting intensity modulated light and a single photodiode (PD) as a receiver. The experiments focus on achieving robustness to cope with unknown received signal strength modifications over time. Therefore, several datasets were collected, where per dataset either the LEDs transmitting power is modified or the PD aperture is partly obfuscated by dust particles. Two relative RSS schemes are investigated. The first scheme uses the maximum received light intensity to normalize the received RSS vector, while the second approach obtains RSS ratios by combining all possible unique pairs of received intensities. The Machine Learning (ML) methods are compared to a relative multilateration implementation. It is demonstrated that the adopted MLP and GP models exhibit superior performance and higher robustness when compared to the multilateration strategies. Furthermore, when comparing the investigated ML models, the GP model is proven to be more robust than the MLP for the considered scenarios.

## 1. Introduction

Determining the location of oneself or mobile objects as accurately as possible has already been a problem of interest for ages. Research efforts to facilitate Location-Based Services (LBS) are being driven by its countless applications and economic potential [1,2]. These applications can be found in industry with for example asset tracking in logistics, automation, and safety. Besides the economic potential in industrial environments, there are also numerous applications for the consumer market. This includes for example navigation assistance and clientele heat maps in retail. Contemporary outdoor localization technologies are dominated by the Global Positioning System (GPS) because of its high accuracy, reliability, and robustness [3]. However, in an indoor setting, GPS performance degrades drastically due to reduced received signal quality and multi path propagation. For indoor scenarios, there is not yet a prevailing technology available. Multiple technologies facilitating indoor positioning are described in the literature [4,5,6] and one way to distinguish between them is that they differ in the physical nature of the signals that are used. The most prominent ones deploy Radio Frequency (RF) [7,8,9], acoustic [10], and light [11] signals.

The fact that Light-Emitting Diodes (LEDs) have become the standard for lighting infrastructure has been a driver for increased research interest in wireless communication systems using visible light signals. LEDs can be switched at a rate much higher than the bandwidth of the human observer allowing to perform wireless communication without compromising the illumination functionality [12]. One such promising technology for indoor localization is Visible Light Positioning (VLP) [13]. It relies on optical wireless communication between modulated or unmodulated [14] artificial visible light sources (typically LEDs) and a receiver sensor.

There are several methods described in the literature [15,16] to derive the position of a mobile receiver using the information that is embedded in these light signals. Classical VLP schemes generally deploy lateration [17], angulation [17], or fingerprinting [18] algorithms to retrieve the coordinates of the receiver. Lateration requires estimations of the distance between the transmitters and receiver. The most prominent strategies to obtain a distance estimate in the context of VLP are based on the Received Signal Strength (RSS) or the Time Difference Of Arrival (TDOA). The angulation approach depends on estimations of the angle of incidence of the transmitted signals. This strategy is generally described as Angle Of Arrival (AOA). The fingerprinting method is based on matching measured data with the contents of a large database that consists of calibration measurements to obtain a location estimate. Depending on the VLP method, the sensor that is selected as a receiver generally is a single Photodiode (PD), an array of photodiodes or because of good anti-interference properties a camera/image sensor [19,20].

The classical VLP schemes have limitations, as often they require close to ideal behavior of the propagation model and its parameters to perform well. Recently, however, it was demonstrated that Machine Learning (ML) techniques like for example Artificial Neural Networks (ANNs) are capable of delivering accurate localization results in the context of VLP [21,22,23,24,25], as they can handle noise naturally and are not bounded by ideal physical behavior. Furthermore, linear interpolation techniques can be used to reduce the amount of required measured data [26]. The adopted ML methods for location estimation in the context of RSS-based VLP typically involve a supervised learning scheme that can be based on measured training data, on data from existing propagation models, or on a mix of measured and propagation model based data [27]. The methods using measured data eliminate the need of exactly characterizing every parameter in the VLP system and eliminate errors introduced due to inaccuracies such as aberrant radiation patterns [28] or flaws in the propagation model [29]. The methods based on a theoretical propagation model require no prior measurements but include these inaccuracies. Moreover, this approach also requires the quantification of the propagation model parameters.

The localization accuracy of an indoor positioning system however is not the sole important parameter that determines its practical applicability in industrial or other commercial use cases. Another equally important characteristic is its robustness or reliability. An important aspect that determines the robustness [27,30] of an indoor positioning system is its ability to cope with a non-static environment where mobile users are to be localized. One has no control over all the ambient parameters that affect the wireless channel and the localization algorithm should be able to compensate for environment dynamics without drastically reducing accuracy. A situation where variation of the surroundings in an RSS based VLP context occurs, is for example in a logistics use case where dust accumulation manifests itself on the surface of the PD because the mobile node (e.g., forklift truck, automated guided vehicle, etc.) is deployed in an industrial area such as a warehouse. The layer of dust partly obscures the effective area of the PD and thus leads to the reduction of the received light intensity.

In this work, an RSS-based VLP approach is considered that uses LEDs as transmitters and a single photodiode as receiving sensor. The focus is on the usage of relative RSS based VLP schemes [31] to provide robustness for varying environmental factors like dust accumulation on the surface of the receiving PD and LED power decrease due to, e.g., aging and dimming. The relative RSS values are used as features in a Multi Layer Perceptron (MLP) and Gaussian Process model (GP), having a 2D location estimate as output. A GP is particularly suitable because it can naturally quantify noise and provide a smooth interpolation behavior. The performance of the ML techniques using relative RSS input features is validated with measurements obtained from an experimental setup. Five measurement sets were acquired to evaluate the robustness of the relative RSS schemes using ML algorithms. The first three sets are measured with different LED currents and thus different emitted optical power. The last two sets were measured with respectively chalk dust and saw dust scattered on the surface of the photodiode. The localization results of the MLP and GP are compared to the results of a relative RSS based multilateration implementation and show high accuracy and robustness.

The main contributions of this work are as follows.

- ML methods featuring GPs and ANNs which use relative intensities are adopted to obtain a more robust RSS based 2D VLP solution. It is shown that relative intensities as inputs for the GP model deliver superior results in terms of accuracy an robustness.
- The impact of unknown received signal strength variations on the ML methods and classical multilateration is extensively studied by conducting experiments where the PD receiver aperture is obfuscated by dust particles and by executing measurements where LEDs are dimmed by reducing the driving currents.

The work is structured as follows. Section 2 describes the system model, where the MLP and GP models are discussed and an analytical derivation of the relative multilateration schemes, which act as the baseline for RSS based VLP methodology, is given. Next, Section 3 illustrates the experimental set-up and discusses the results by comparing the performance of the ML models and the multilateration strategies. Section 4, the conclusion, summarizes the most important results.

## 2. System Model

### 2.1. Propagation Model

In this work, a VLP set-up is considered consisting of four LEDs mounted in a horizontal plane at a height *h* above the observation plane in which a mobile node is localized using a photodiode (PD) as receiver. An individual LED *l* in the set-up transmits a power-switched optical intensity modulated waveform according to

(1) $$s_{l}\left(t\right)=\frac{P_{t}}{2}\left[1+\mathrm{sgn}\left(\sin\left(2\pi f_{0}2^{l}t+\phi_{l}\right)\right)\right].$$

Here, *l*ϵ$\left\{0,1,2,3\right\}$, $P_{t}$ is the transmitted optical power, $f_{0}$ is the base frequency, and $\phi_{l}$ is a random and unknown phase offset which is different for every LED in the system. The PD mounted on the mobile node receives the sum of the channel attenuated transmitted signals $s_{l}\left(t\right)$, which is denoted by r(t) and given by [32]

(2) $$r\left(t\right)=\sum_{l=0}^{L-1}\alpha_{l}R_{p}s_{l}\left(t\right)+\beta +n\left(t\right).$$

where $R_{p}$ is the responsivity of the PD, *L* is the total number of LEDs, β is a DC contribution of ambient light sources, n(t) is the noise component, and $\alpha_{l}$ is the channel attenuation [33]:

(3) $$\alpha_{l}=\frac{A_{r}}{d_{l}^{2}}G_{l}\left(\theta_{l},\psi_{l}\right)\cos\gamma_{l},$$

where $A_{r}$ is the effective area of the PD and $d_{l}$ is the euclidean distance between an LED *l* and the PD, while $G_{l}\left(\theta_{l},\psi_{l}\right)$ describes the angular distribution of radiant output power of each LED *l*, where $\theta_{l}$ is the inclination angle and $\psi_{l}$ the azimuthal angle. $\gamma_{l}$ is the angle of incidence at the receiver. No optical filter or concentrator were used in the setup. At the PD, the signal can be demultiplexed because of the Frequency division Multiple Access (FDMA) approach by assigning LED transmitting frequencies as in Equation (1) [34]. The channel attenuation for every LED is extracted by evaluating the amplitude and phase spectrum in the frequency domain, at the associated frequency bins.

In this work, environmental dynamics are simulated by changing transmitter LED currents and by scattering dust on the surface of the PD. The effect of dust accumulation on the received light intensity is assumed to be equal for every LED’s contribution. It can thus be modeled by adding an extra factor $\eta_{r}\leq 1$ in the expression of the received light intensity in Equation (2) leading to Equation (4)

(4) $$r\left(t\right)=\sum_{l=0}^{L-1}\eta_{r}\alpha_{l}R_{p}s_{l}\left(t\right)+\beta +n\left(t\right).$$

At a certain location (x,y) after demultiplexing of the received signal, a set of L=4 intensities $r=(r_{0},r_{1},r_{2},r_{3})$ is retrieved. In this work, two methods to obtain relative intensities from the set *r* of absolute intensities are investigated. In the first approach, the relative signal strength $r_{rel,max}$ is obtained by dividing *r* by the maximum value of *r*, thus resulting in three relative intensities and intensity 1 at the index of the received maximum. The second approach considers all possible unique paired combinations that can be drawn from the set *r*. This results for L=4 in 42=6 pairs, where the vector of the ratios of the pairs yields $r_{rel,all}$. The relative intensities are then used as input features in an MLP model, GP model, and relative multilateration scheme, generating a location estimate $\left(\hat{x},\hat{y}\right)$. The accuracy of the obtained results is evaluated by using the euclidean distance error metric:

(5) $$E_{p}=\sqrt{{\left(\hat{x}-x\right)}^{2}+{\left(\hat{y}-y\right)}^{2}},$$

where (x,y) are the ground truth coordinates. The models described in the coming sections will take the relative or absolute intensities as features and the coordinates as outputs. The following ML models were selected because they are naturally suited to model noisy data and exhibit smooth behavior, in line with the physical properties of the system under consideration.

### 2.2. Multilayer Perceptron

**Figure 1:**
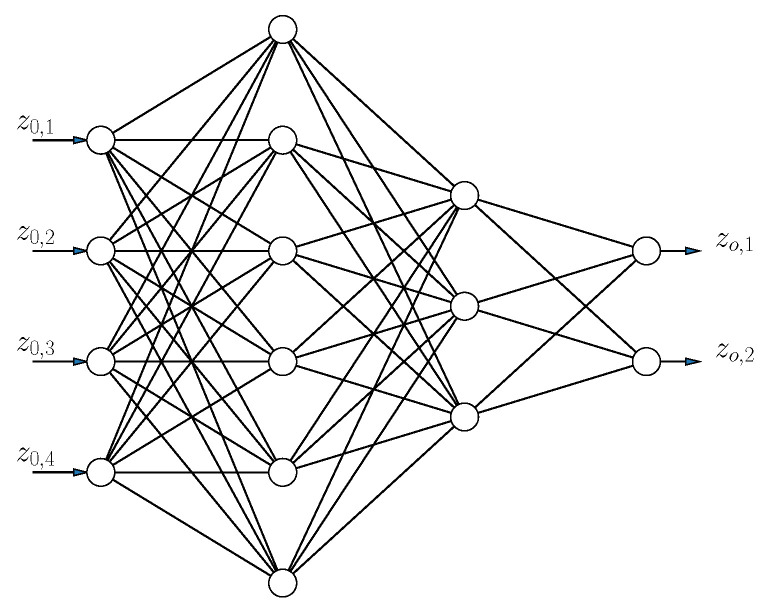
Structure of an Artificial Neural Network (ANN), which consists of multiple nonlinear transformations $\sigma (W_{i}z_{i-1})$ of the inputs $z_{0}$.

When using physical models, it is not always straightforward how to deal with noise, unknown (non-Lambertian) radiation patterns, obstruction of field of view, and dust. This is where data-driven approaches can offer advantages. One of these models, used in the context of VLP, is the MLP model [21,22], of which the structure is shown in Figure 1. An MLP consists of at least three layers: an input layer (represented by vector $z_{0}$) containing the features (relative intensities), one or more hidden layers (represented by vector $z_{i}$), and an output layer (represented by vector $z_{o}$) (predicting the position). Each layer is a transformation of the previous layer:

$$z_{i}=\sigma \left(W_{i}z_{i-1}+b_{i}\right).$$

Here, $W_{i}$ is a matrix (called the weights), $b_{i}$ is a vector (called the bias), and σ is an activation function that is nonlinear except for the last layer. The activation function that is used here is the hyperbolic tangent function. The sizes of the hidden layers are hyperparameters that are chosen upfront or through cross-validation.

The weights $W_{i}$ have to be optimized (trained) by minimizing a loss function. In this case, the loss function is simply $E_{p}^{2}$. To update the weights every iteration, the fast converging Levenberg–Marquardt algorithm [35] is used. One of the drawbacks of this method is that it uses the Jacobian matrix, which increases the computational complexity rapidly with neural network size. Additionally, in larger datasets minibatching cannot be combined with this optimization strategy [36].

### 2.3. Gaussian Processes

One drawback of MLPs is that they generally do not perform well when using small datasets, and regularization is not obvious without hyperparameter tuning. The GP [37] is naturally suited to tackle these issues. A Gaussian Process is a probabilistic generalization of an MLP with one hidden layer, and assumes that the prior on the weights of the last layer are Gaussian. When taking the limit to an infinite amount of nodes (dimension of hidden layer), it is possible to use the kernel trick [38], which defines the GP.

More formally a GP is defined by a kernel function $k(z,z^{\prime })$ and a mean function μ(z) such that the probability of any finite set of input–output pairs ${\left\{\left(z_{i},f_{i}\right)\right\}}_{i=1}^{N}$ is distributed according to a Gaussian distribution with a mean ${\left[\mu \left(z_{i}\right)\right]}_{i=1}^{N}$ and a covariance *K*. $f_{i}$ are vectors containing the euclidean coordinates. Each entry of the covariance is defined by $K_{ij}=k\left(z_{i},z_{j}\right)$. The mean function is considered to be zero everywhere in this work.

The kernel function that is chosen here is isotropic because of the approximate symmetry of the problem, more specifically, the Radial Basis Function (RBF) kernel is used:

(6) $$k\left(z,z^{\prime }\right)=\sigma_{k}^{2}\exp\left(\frac{∥z-z^{\prime }{∥}^{2}}{2\Delta^{2}}\right).$$

The hyperparameters $\zeta =\{\sigma_{k},\Delta \}$ are determined by maximizing the log likelihood of the data:

(7) $$\begin{array}{cc} \hat{\zeta } & =\arg\max_{\zeta }\log p\left(f|\zeta \right) \end{array}$$

(8) $$\begin{array}{cc} \; & =\arg\max_{\zeta }-\frac{1}{2}\left(\log|2\pi K|+f^{T}K^{-1}f\right) \end{array}$$

It is possible to obtain a prediction for a new input $z_{★}$ by computing the conditional distribution, which results in a Gaussian distribution with mean $k_{★}^{T}K^{-1}f$ and covariance $k_{★★}-k_{★}^{T}K^{-1}k_{★}^{T}$, where

(9) $$\begin{array}{cc} {\left(k_{★}\right)}_{i} & =k(z_{★},z_{i}), \end{array}$$

(10) $$\begin{array}{cc} k_{★★} & =k(z_{★},z_{★}). \end{array}$$

**Figure 2:**
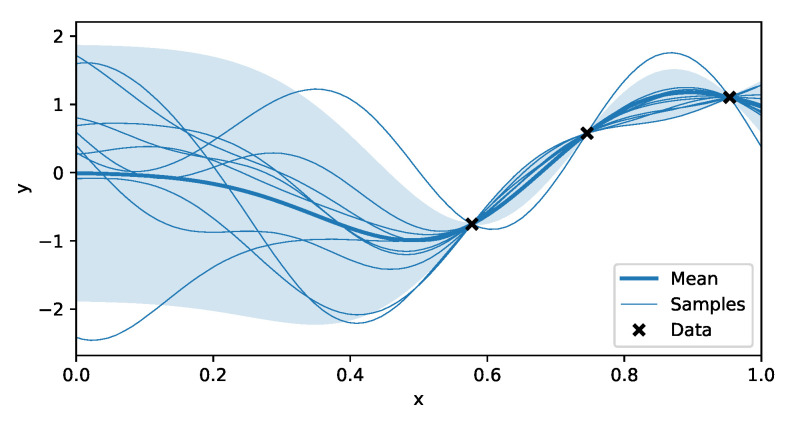
Predictions made by a Gaussian Process (GP). The data used to train the GP model are indicated with the black markers. The predictive distribution consists of a mean, indicated with the thick blue line, and a covariance of which the diagonal elements are used to calculate the 95% confidence interval plotted around it. Since the prediction consists of a correlated Gaussian, it is possible to sample from this distribution, as shown in the plot. Every one of those samples possibly match with the real, underlying function.

Thus, additionally this provides a variance on the prediction, which can potentially be used to quantify how certain we are about the position. This predictive distribution is illustrated in Figure 2.

### 2.4. Relative RSS Based Multilateration Scheme

In general, a planar multilateration problem is solved by finding a solution for the following set of *L* equations, given by Equation (11) where $\left(\hat{x},\hat{y}\right)$ is the unknown position of the mobile receiver.

(11) $$\begin{array}{cc} {\left(\hat{x}-x_{l}\right)}^{2}+{\left(\hat{y}-y_{l}\right)}^{2}+h^{2} & =d_{l}^{2},\;l=0,..,L-1. \end{array}$$

The coordinates $\left(x_{l},y_{l}\right)$ with l∈0,⋯,L−1 (L≥2) are the fixed locations of the LED beacons and the parameter $d_{l}$ is the euclidean distance between the LEDs and the receiver. In this work, the distance estimation is based on the received signal strength [39]. For example, dust accumulation on the surface of the PD will reduce the received light intensity, resulting in an erroneous distance estimation $\delta_{l}=\frac{d_{l}}{\sqrt{\eta_{r}}}$. This link can be found by comparing the received intensities $r_{d}$ leading to $d_{l}$ and $r_{\delta }$ leading to $\delta_{l}$ using Equation (2) (after demultiplexing) and Equation (3), where it is given that $r_{\delta }=\eta_{r}r_{d}$. The aforementioned leads to a set of *L* equations:

(12) $$\begin{array}{cc} {\left(\hat{x}-x_{l}\right)}^{2}+{\left(\hat{y}-y_{l}\right)}^{2}+h^{2} & =\delta_{l}^{2},\;l=0,..,L-1. \end{array}$$

The first relative RSS multilateration scheme employed in this work normalizes the received intensities with respect to the maximum intensity in a received set *r*. The equation featuring the smallest estimated distance $\delta_{l}$, and thus the maximum received intensity, is divided by the equations defined in Equation (11). More formally the equation with distance $\delta_{k}=\min\left\{\delta_{0},\;\delta_{1},\;\cdots ,\;\delta_{L-1}\right\}$ is used to obtain a new set of equations:

(13) $$\begin{array}{cc} \frac{{\left(\hat{x}-x_{k}\right)}^{2}+{\left(\hat{y}-y_{k}\right)}^{2}+h^{2}}{{\left(\hat{x}-x_{l}\right)}^{2}+{\left(\hat{y}-y_{l}\right)}^{2}+h^{2}} & ={\left(\frac{\delta_{k}}{\delta_{l}}\right)}^{2}=c_{l},\;l=0..,k-1,k+1,..,L-1. \end{array}$$

As a result of this operation, the unknown attenuation $\eta_{r}$ is no longer present in the equation set to be solved. If one selects any equation from Equation (13) with $c_{n}$ on the right hand side and rearranges the terms the following relationship is found,

(14) $$\begin{array}{c} \begin{array}{cc} \; & {\hat{x}}^{2}\left(1-c_{n}\right)-2\hat{x}\left(x_{k}-c_{n}x_{n}\right)+x_{k}^{2}-c_{n}x_{n}^{2}+\\ \; & {\hat{y}}^{2}\left(1-c_{n}\right)-2\hat{y}\left(y_{k}-c_{n}y_{n}\right)+y_{k}^{2}-c_{n}y_{n}^{2}+\\ \; & h^{2}\left(1-c_{n}\right)=0, \end{array} \end{array}$$

Which finally leads to

(15) $$\begin{array}{c} {\left(\hat{x}-u_{n}\right)}^{2}+{\left(\hat{y}-v_{n}\right)}^{2}=\rho_{n}^{2}, \end{array}$$

with

(16) $$\begin{array}{c} \begin{array}{cc} u_{n} & =\frac{x_{k}-c_{n}x_{n}}{1-c_{n}},\\ v_{n} & =\frac{y_{k}-c_{n}y_{n}}{1-c_{n}},\\ \rho_{n}^{2} & =\frac{c_{n}}{1-c_{n}}R_{n}^{2}-\frac{1}{1-c_{n}}R_{k}^{2}+u_{n}^{2}+v_{n}^{2}, \end{array} \end{array}$$

and

(17) $$\begin{array}{c} \begin{array}{cc} R_{n}^{2} & =(x_{n}^{2}+y_{n}^{2}+h^{2}),\\ R_{k}^{2} & =(x_{k}^{2}+y_{k}^{2}+h^{2}) \end{array} \end{array}$$

and

n=0,..,k−1,k+1,L−1

This approach results in a new set of L−1 equations:

(18) $$\begin{array}{cc} {\left(\hat{x}-u_{i}\right)}^{2}+{\left(\hat{y}-v_{i}\right)}^{2} & =\rho_{i}^{2},\;i=0..,k-1,k+1,..,L-1. \end{array}$$

Remark that this set of equations is again of the same form as the general multilateration set. However, one equation has been sacrificed to eliminate unknown additional attenuation parameter $\eta_{r}$.

The second relative localization scheme employed in this work uses all possible unique combinations which can be selected from the set of *L* equations, defined by Equation (11), and is given by L2. This is a variation of the first scheme. Every combination (i,j) results in an equation as in Equation (15) where

(19) $$\begin{array}{c} \begin{array}{cc} c_{i,j} & ={\left(\frac{\delta_{i}}{\delta_{j}}\right)}^{2}\\ u_{i,j} & =\frac{x_{i}-c_{i,j}x_{j}}{1-c_{i,j}}\\ v_{i,j} & =\frac{y_{i}-c_{i,j}y_{j}}{1-c_{i,j}}\\ \rho_{i,j}^{2} & =\frac{c_{i,j}}{1-c_{i,j}}R_{i,j}^{2}-\frac{1}{1-c_{i,j}}R_{i}^{2}+u_{i,j}^{2}+v_{i,j}^{2}. \end{array} \end{array}$$

The location estimate $\left(\hat{x},\hat{y}\right)$ of the above-described analytical multilateration method can finally be obtained with least squares optimization [40]. The solution adopted in this work employs a non-linear least squares approach, using the Broyden–Fletcher–Goldfarb–Shanno algorithm (BFGS) [41], where the objective is to minimize the cost function $J(\hat{x},\hat{y})$ given by Equation (20) using Equation (19) with *p* the number of available equations.

(20) $$\begin{array}{c} J\left(\hat{x},\hat{y}\right)=\frac{1}{p-1}\sum_{i=0}^{p-1}{\left(\hat{\rho_{i}}-\rho_{i}\right)}^{2}. \end{array}$$

## 3. Measurements and Results

### 3.1. Experimental Set-Up

**Figure 3:**
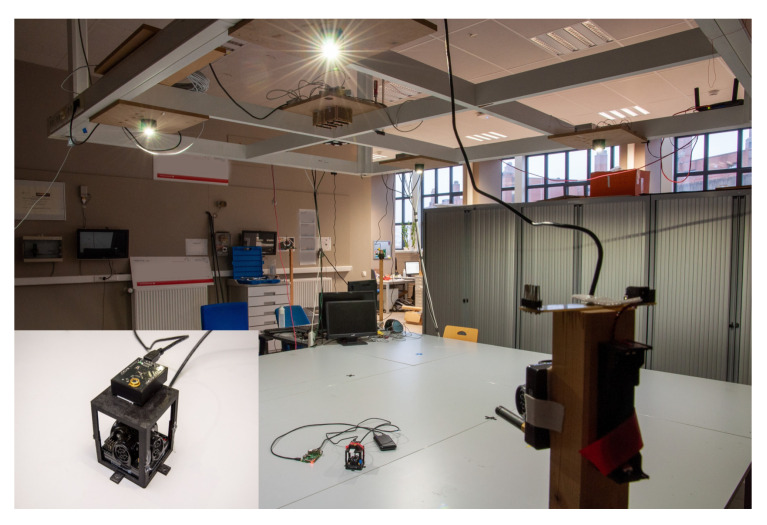
A photo of the complete experimental set-up with four light-emitting diodes (LEDs) at height h=1.284 m.

**Figure 4:**
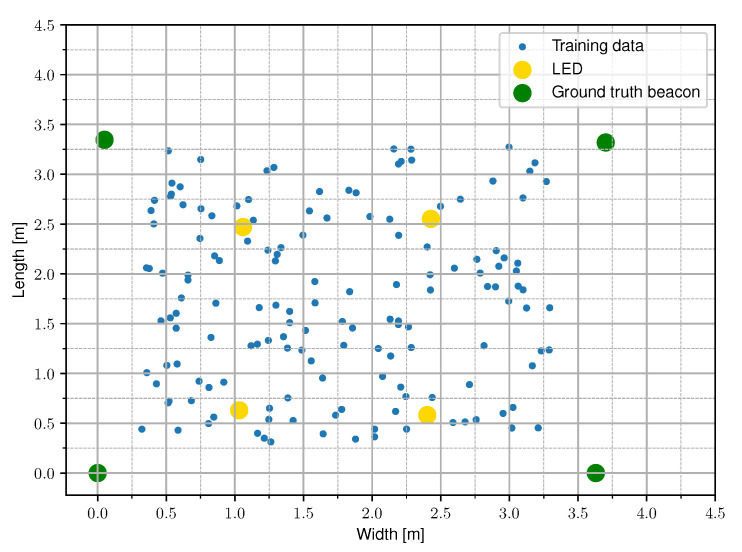
A conceptual top view of the setup with four LEDs at a height *h*. The measured data from the experimental set-up is sampled randomly in the receiver plane.

**Figure 5:**
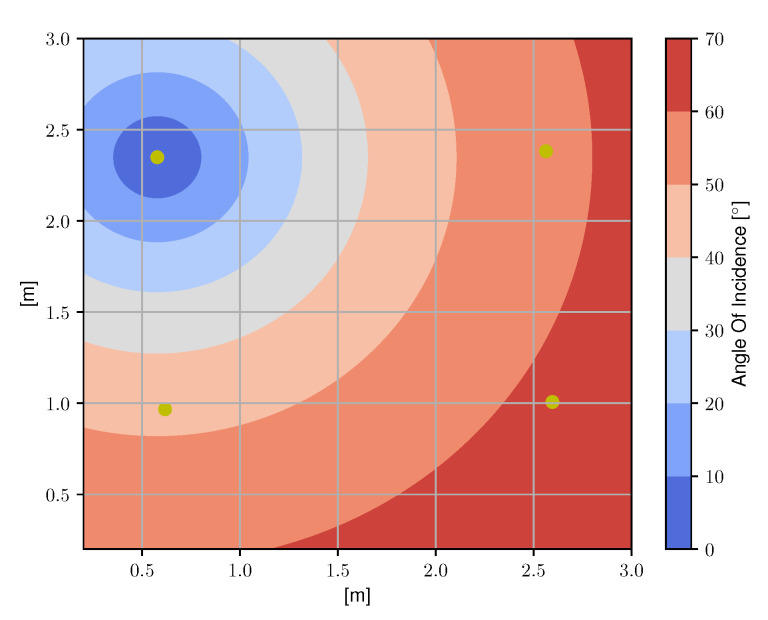
A top view of the set-up with the angles of incidence $\gamma_{l}$ at the photodiode (PD) highlighted for a single LED. The wide range of angles of incidence results in a representative experimental set-up.

**Figure 6:**
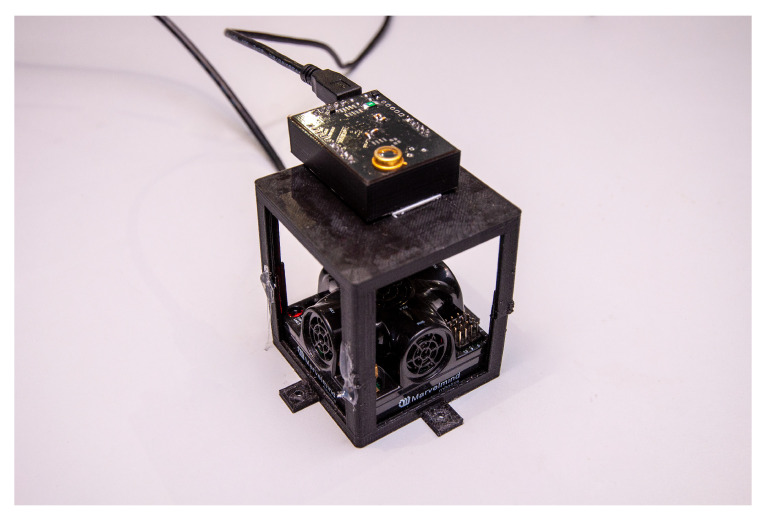
Image of the custom designed receiver in the experimental setup, the device on the top is the Visible Light Positioning (VLP) receiver and the device on the bottom is the mobile node of the acoustic ground truth system (Marvelmind Robotics Super-NIA-3D).

To evaluate the performance of the Relative RSS-based VLP schemes, an experimental setup was constructed. An image of the setup is depicted in Figure 3 and a top view is shown in Figure 4. It consists of 4 LEDs (Bridgelux BXRE-C3001-D24, Driver: Analog Devices LTM-8005 and board DC2257A) in a quasi rectangular set-up at a height of h=1.284 m and a PD (Thorlabs FDS100 Si Photodiode)-based custom receiver that can move in a plane of 3 m × 3 m, thus covering a wide range of angles of incidence $\gamma_{l}$ at the receiver, as is depicted in Figure 5. The receiver module is depicted in Figure 6. The main parameters of the setup are summarized in Table 1.

**Figure 7:**
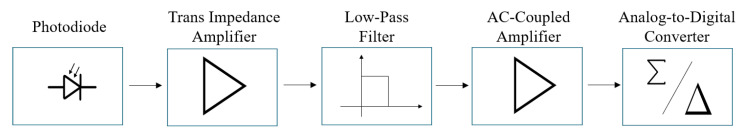
A block schematic representation of the receiver circuit.

The receiver features a Programmable System On Chip (PSOC)(Cypress Semiconductor PSOC 5lp, Component: CY8C5868LTI-LP039) that implements most of the analog signal processing on-chip and has a high resolution Sigma-Delta Analog-to-Digital Converter (ADC)(On-chip component PSOC) for signal digitization. A block schematic representation of the receiver circuit is given in Figure 7.

The photo current generated by the photodiode is coupled into a Transimpedance Amplifier (TIA)(On-chip component PSOC) to be converted to a voltage. Next, the output of the TIA is coupled into a passive first-order Low-Pass Filter (LPF). The output signal of the LPF is AC-coupled to remove the ambient light and DC-biased at 50% of the ADC range because the circuit operates with single positive supply components. The signal is then used as input in a high gain amplifier with offset compensation. This amplified signal is coupled into the ADC where it is converted to the digital domain with a precision of 14 bit. A summary of the receiver circuit parameters is given by Table 2.

After ADC conversion, the measured samples are first buffered in memory and then transferred via Direct Memory Access (DMA) to the ARM (https://developer.arm.com/ip-products/processors/cortex-m/cortex-m3) Cortex M3 Micro Controller Unit (MCU) on the PSOC chip where a hardware interrupt is triggered when the DMA-transfer is completed. Next, the software on the MCU handles the data transfer to the connected Raspberry Pi (https://www.raspberrypi.org/products/raspberry-pi-3-model-b/) SBC over a USB interface.

**Figure 8:**
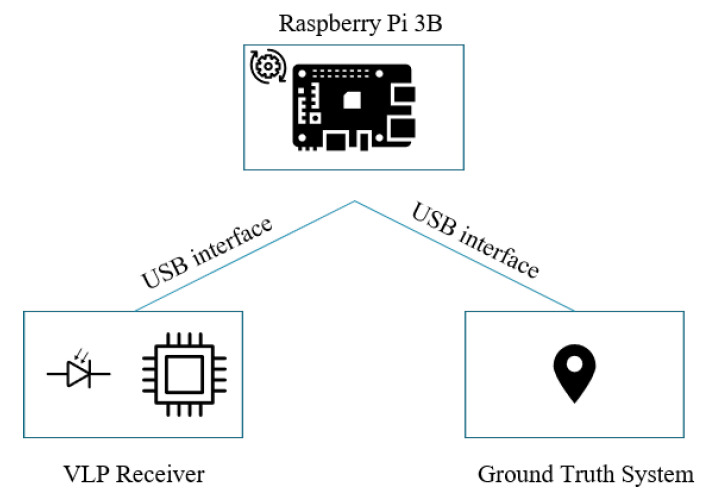
A block schematic representation of the receiver on a system level.

A highly accurate (±2 cm differential) acoustic ground truth system(Marvelmind Super-NIA-3D) is used to acquire the coordinates of the measurement locations. Both the ground truth system and the custom VLP receiver are interfaced with a Single Board Computer(Raspberry Pi 3B) (SBC) which simultaneously acquires the coordinates of the measurement locations and the associated light intensities as depicted in Figure 8.

In this work, the SBC is only used for signal demultiplexing and data logging but it can easily be configured to deliver real time location updates based on the measured received light intensities.

**Figure 9:**
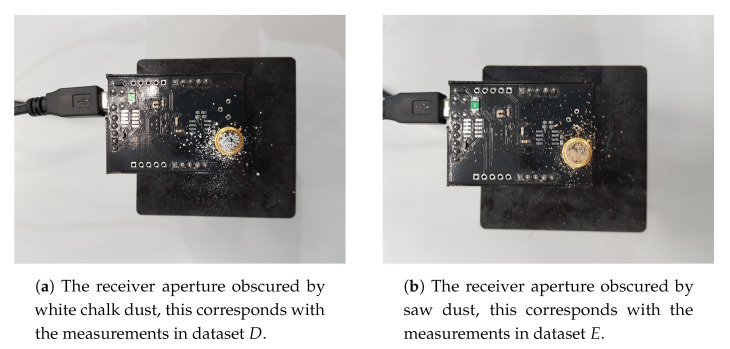
Dust particles scattered on the aperture on the receiver, representing possible changes in the environment during operation of the VLP system where robustness is required.

During the experimental evaluation, the following measurements were executed. In total, five datasets are acquired by randomly moving in the horizontal observation plane, both inside and outside the rectangular frame composed by the LEDs, logging ground truth location coordinates and linked light intensities. The first three sets were measured with different LED driving currents, which are 250 mA (Set *C*), 300 mA (Set *B*), and 350 mA (Set *A*). The last two sets were measured at a LED current of 350 mA with chalk dust (Set *D*) and saw dust (Set *E*) randomly scattered onto the PD surface area thus obscuring the PD aperture as is shown in Figure 9. Set *A* is to be considered as the installation configuration. The LEDs are transmitting the Table 3 gives an overview of the nature and the size of the measured datasets and provides a reference name for each set.

The receiver configuration parameters in terms of signal processing and ADC parameters is the same for every dataset. For both machine learning algorithms, three models are trained with set *A*. One model is trained using the absolute RSS values *r*, while the other two other models are trained using the relative intensity schemes $r_{rel,max}$ and $r_{rel,all}$. For the absolute RSS case, the models are trained with a subset of set *A* and their performance is evaluated on an independent validation set, sampled from set *A*. For the relative RSS cases, the models are trained with set *A* and the performance is determined using the other datasets. For the ML models trained with relative intensities, the size of the training set (set *A*) is varied as well to compare their performance in terms of data efficiency, where data efficiency is defined as model performance per number of training data points. The models are tuned separately for each different training set. The MLP and GP model performances are finally compared to the relative RSS multilateration implementations.

### 3.2. Localization Results

#### 3.2.1. Performance Comparison Relative vs. Absolute Intensities as Features

**Figure 10:**
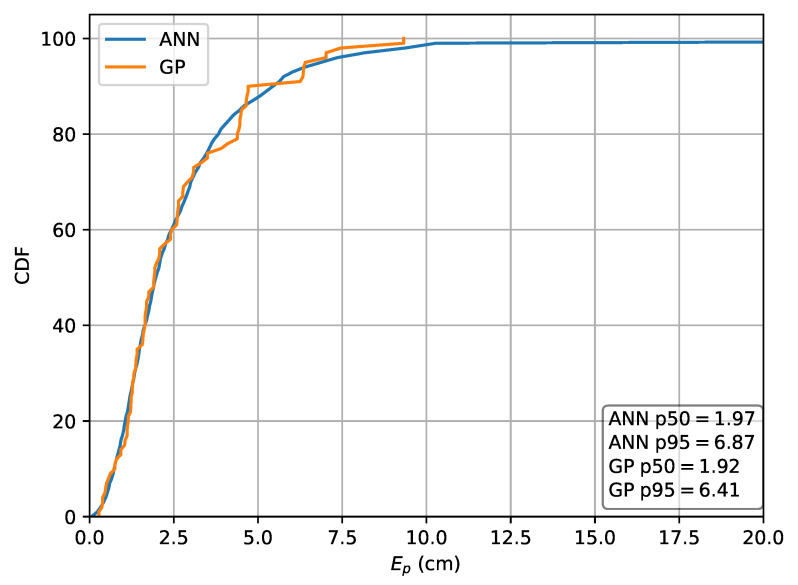
Cumulative distribution of the error evaluated on set *A* for the Machine Learning (ML) methods in the case the absolute intensities *r* are used as features.

First, the performance of the ML methods where relative intensities are used as features is investigated and compared to the absolute RSS case. To achieve this, dataset *A* is divided in a training set (60%) and a validation set (40%) and the localization accuracy obtained from the validation set is used as the performance metric. When the absolute RSS values are used as input features in the MLP and GP models, the MLP results in a p50 localization error of 1.99 cm and a p95 localization error of 7.17 cm while the GP results in a p50 error of 1.92 cm and a p95 error of 6.41 cm. The Cumulative Distribution Function (CDF) of the localization error in the absolute RSS case is depicted in Figure 10.

**Figure 11:**
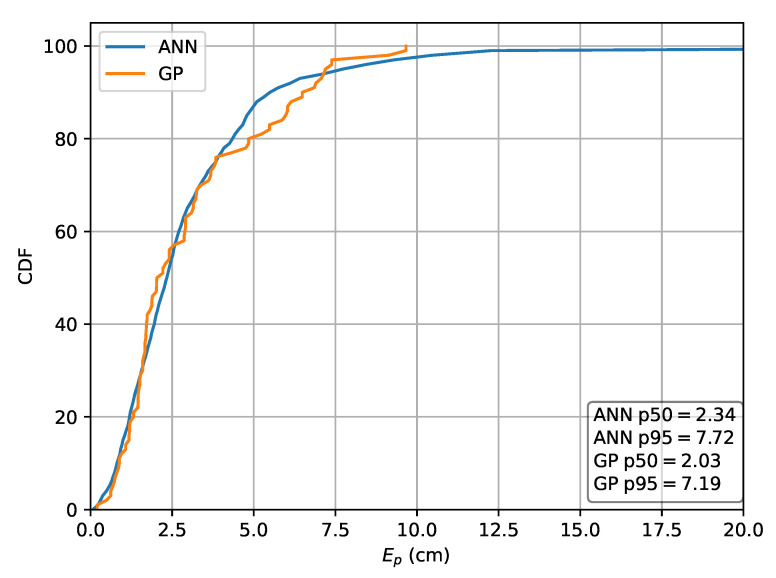
Cumulative distribution of the error evaluated on set *A* for the ML methods in the case the relative intensities $r_{rel,max}$ are used as features.

Next, the relative RSS scheme using $r_{rel,max}$ to obtain the input features is investigated. For this scheme, the MLP results in a p50 localization error of 2.36 cm and a p95 error of 7.41 cm, while the GP results in a p50 localization error of 2.03 cm and a p95 error of 7.19 cm. The CDF of the localization error for this scheme is depicted in Figure 11.

**Figure 12:**
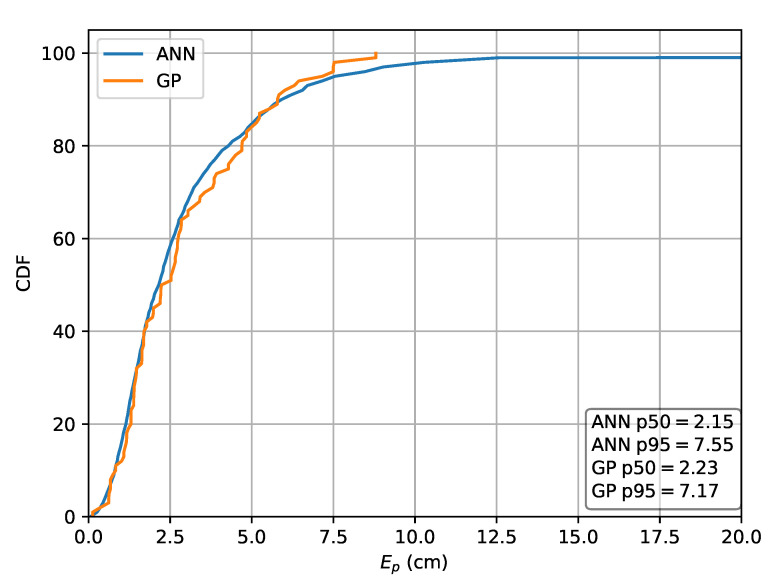
Cumulative distribution of the error evaluated on set *A* for the ML methods in the case the relative intensities $r_{rel,all}$ are used as features.

The second relative RSS approach uses $r_{rel,all}$ to obtain input features. For this strategy, the MLP results in a p50 localization error of 2.13 cm and a p95 error of 7.37 cm while the GP results in a p50 error of 2.35 cm and a p95 error of 6.86 cm. The CDF of the localization error for this scheme is depicted in Figure 12.

**Figure 13:**
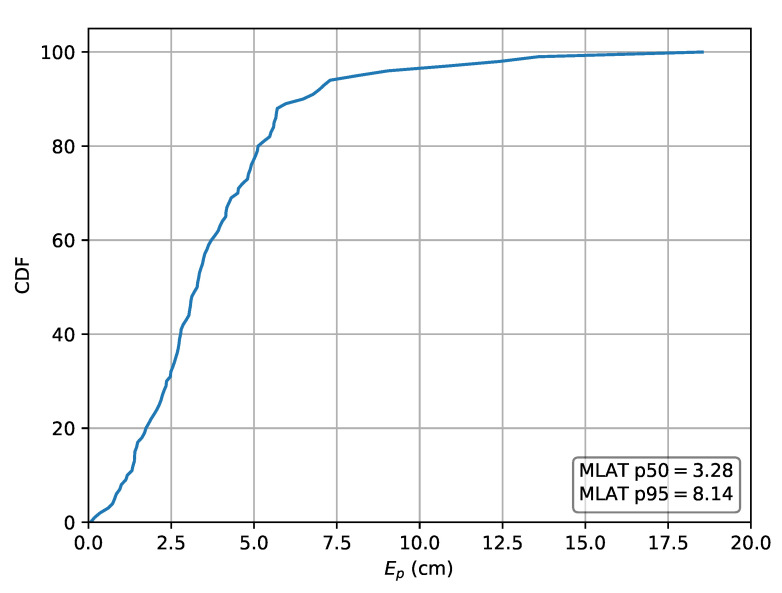
Cumulative distribution of the error evaluated on set *A* for the absolute multilateration scheme using *r* as input.

**Figure 14:**
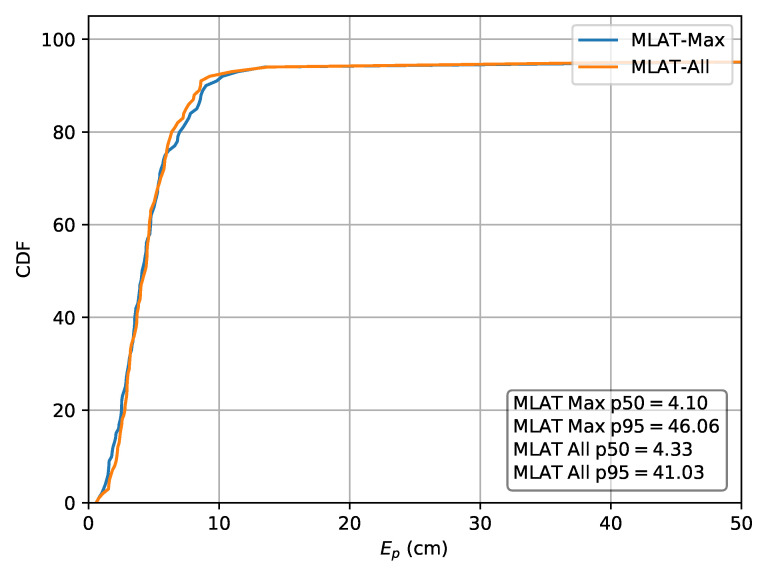
Cumulative distribution of the error evaluated on set *A* for the relative multilateration schemes using $r_{rel,max}$ and $r_{rel,all}$.

For comparison, both the relative multilateration schemes and an absolute implementation are also applied to dataset *A*. In the case the absolute values are used, this results in a p50 localization error of 3.28 cm and a p95 error of 8.17 cm. Next, the relative scheme using $r_{rel,max}$, results in a in a p50 localization error of 4.09 cm and a p95 error of 46.06 cm. The second scheme, using $r_{rel,all}$, results in a p50 error of 4.33 cm and a p95 error of 41.03 cm. The CDF of the localization error for the absolute approach and both relative lateration schemes are respectively depicted in Figure 13 and Figure 14. The p50 and p95 values are summarized in Table 4.

If one compares the results of the ML models to the multilateration approach when relative intensities are used, one can see that GP and MLP both outperform the analytical lateration strategy. Specifically, the p95 performance of the ML models is about 6x better than that of the multilateration strategy which makes the ML models superior candidates to achieve a more robust localization solution as will be demonstrated in Section 3.2.2. From these results one can also conclude that using relative intensities as input features in the MLP and GP models, performs similarly well as in the case with the absolute intensities used as features.

#### 3.2.2. Cross-Validation between Datasets

To demonstrate the robustness of the relative RSS schemes, the ML models that are trained with set *A* are cross-validated with the remaining datasets. Furthermore, to assess the data efficiency, the size of the training set is varied to be N∈(50,75,100,125,177), where the last value is the full size of set *A*. For reference, the p50 and p95 localization results of a classical RSS-based multilateration implementation are included in Table 5. The classical RSS-based lateration solution uses absolute intensities and does not account for changes in the environment in any way. It can be seen from Table 5 that the localization accuracy deteriorates drastically when there is no mechanism that compensates the modified environment.

For the ML models, the relative RSS scheme using $r_{rel,max}$ is investigated first and compared to the performance of the second relative RSS scheme, where $r_{rel,all}$ is used as input features. The results in terms of the p50 and p95 localization accuracy are summarized in Table 6 for the GP models and in Table 7 for the MLP model.

If one observes the rows *B* and *C* From Table 6, one can see that the GP model is capable of delivering reliable positioning results, even in the case when there is only a small amount of training data available. For set *B* at N=50, the GP already performs well with a p50 localization error of 5.30 cm and a p95 error of 19.45 cm. Increasing the training set to N=176, being the full size of set *A*, improves the P95 error significantly to 11.52 cm and the p50 error to 3.61 cm. The localization accuracy of the GP model cross validated on set *C*, where the LEDs are further dimmed, is negatively impacted for both the p50 and p95 values. The best results are found when N=176 with a p50 localization error of 7.77 cm and a p95 error of 18.39 cm.

It should, however, be noted that the receiver parameters are unchanged for all measured datasets, thus resulting in non-ideal gain settings for the lower light intensity level corresponding with set *C*. It is expected that employing an adaptive gain control scheme to optimize the dynamic range usage of the ADC would again improve the results, while because of the use of relative intensities as features, no model retraining would be required. Datasets *D* and *E* represent possible environment dynamics in industrial logistics environments where dust accumulation could obscure the receiver aperture. For set *D*, where chalk dust is scattered on the PD aperture, the best performance for the GP model is again achieved when N=176 with a p50 localization error 9.99 cm and a p95 error of 20.53 cm. Finally, for set *E* where saw dust is scattered on the PD surface, the GP model remains capable of providing robust positioning results with a best case p50 error of 13.88 cm and a p95 error of 22.95 cm. When one differentiates between the LED dimming scenarios *B* and *C* and the aperture obfuscation situations *D* and *E*, one can see that the p50 and p95 errors are higher in the latter cases. A possible cause for the increase in error is that the aperture blockage factor $\eta_{r}$ of the PD with dust particles is not fully independent of the angle of incidence $\gamma_{l}$.

**Figure 15:**
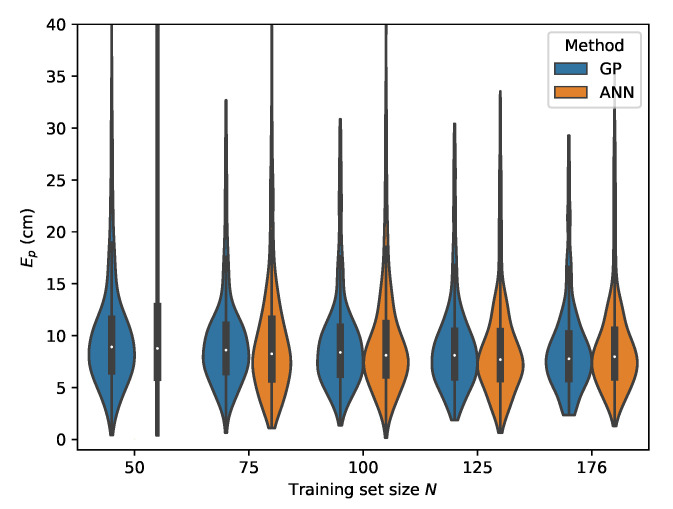
Cross-validation with set *C* where the $r_{rel,max}$ input features are used.

**Figure 16:**
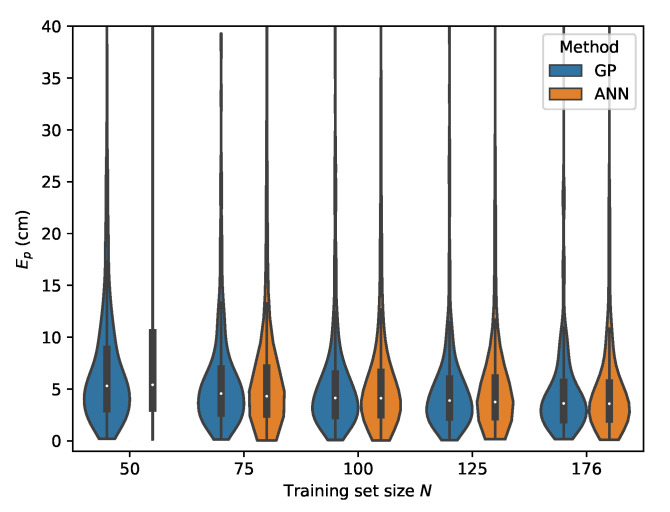
Cross-validation with set *B* where the $r_{rel,max}$ input features are used.

**Figure 17:**
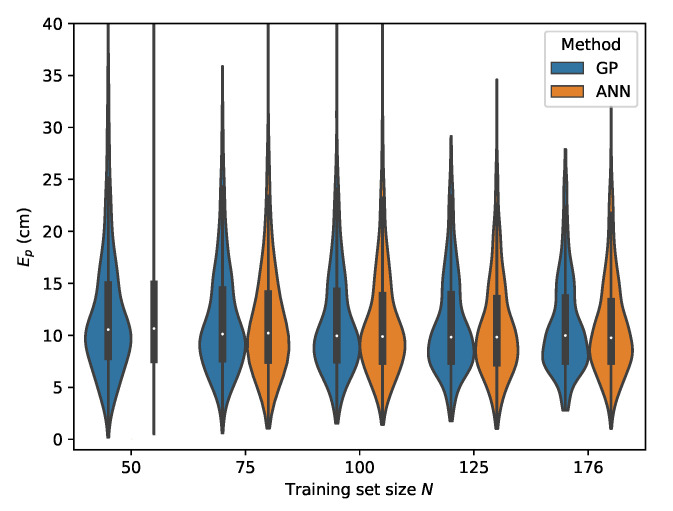
Cross-validation with set *D* where the $r_{rel,max}$ input features are used.

**Figure 18:**
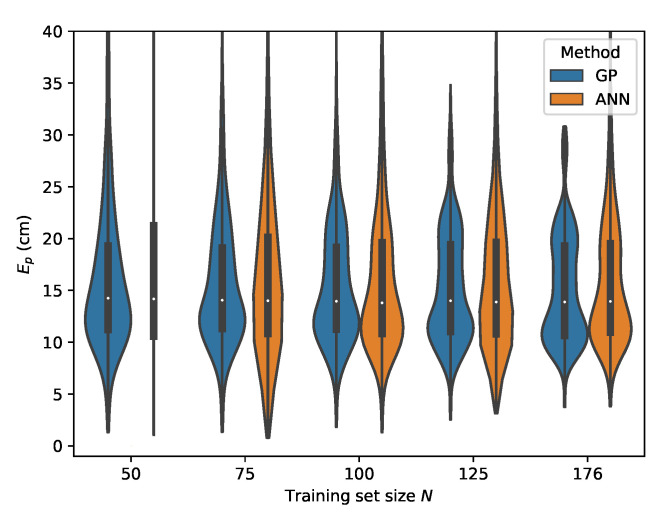
Cross-validation with set *E* where the $r_{rel,max}$ input features are used.

From Table 7, it can be seen that the MLP model is also capable of delivering reliable localization results in a non-static environment. However, when comparing the performance of the GP and MLP models, it can be seen that the GP is much more data efficient than the MLP model. The P95 localization error in the lower training data regimes is much higher in the case of the MLP. This effect decreases with increasing training set size and at N=176 the MLP and GP deliver similar results, with the difference that the GP model provides slightly more robust localization. This can be observed by considering the p50 and p95 values in Table 6 and Table 7 where N=176. The error distributions for all datasets, examining the ML models performance in function of the dataset size, are given in Figure 15, Figure 16, Figure 17 and Figure 18.

The results for the second relative RSS scheme are summarized in Table 8 for the GP and in Table 9. Similar conclusions can be drawn for this scheme when comparing the GP model with the MLP model. Both models succeed in delivering robust localization with a worst case result occurring when cross validating with set *E*, yielding a p50 and p95 error of 13.66 cm and 25.52 cm, respectively, for the GP and a p50 and p95 error of 13.82 cm and 27.55 cm, respectively, for the MLP.

**Figure 19:**
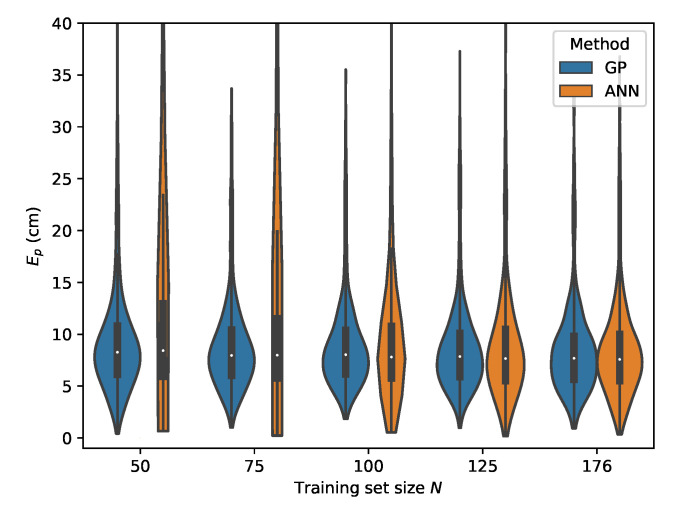
Cross-validation with set *C* where the $r_{rel,all}$ input features are used.

**Figure 20:**
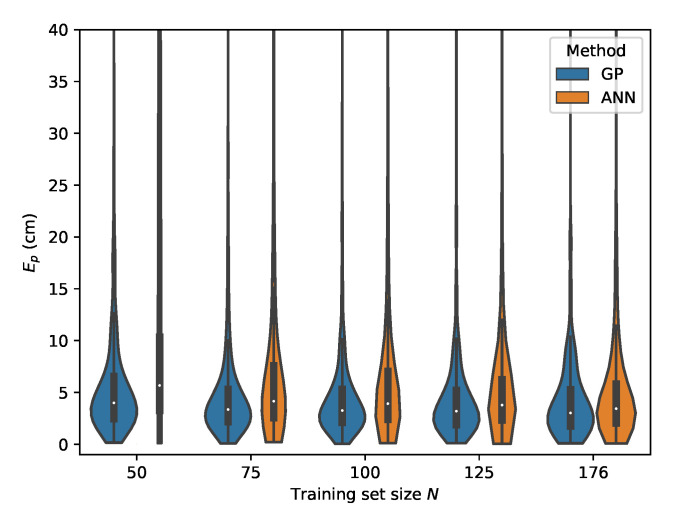
Cross-validation with set *B* where the $r_{rel,all}$ input features are used.

**Figure 21:**
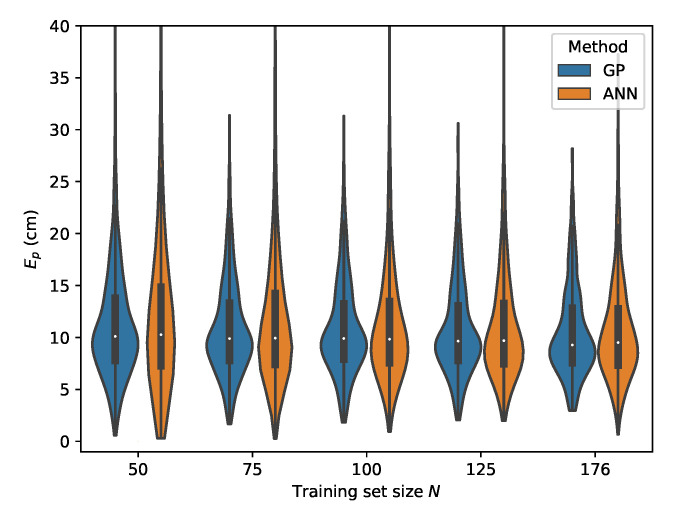
Cross-validation with set *D* where the $r_{rel,all}$ input features are used.

**Figure 22:**
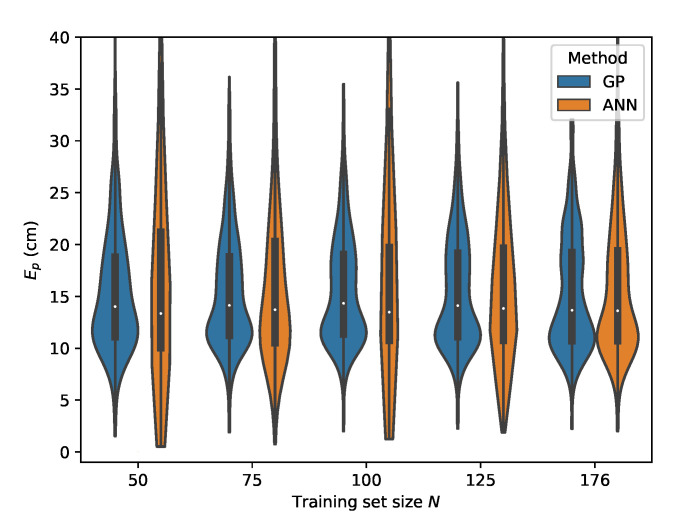
Cross-validation with set *E* where the $r_{rel,all}$ input features are used.

The error distributions for all datasets, comparing the ML models performance in function of the dataset size, are given in Figure 19, Figure 20, Figure 21 and Figure 22.

If one analyzes the performance of the ML models for both the relative RSS schemes, one can conclude that both schemes lead to accurate localization and increased robustness but the second scheme based on $r_{rel,all}$ overall, slightly outperforms the first scheme.

When the cross-validation results for the ML schemes are compared to the outcome of the classical multilateration algorithm from Table 5, one can see that the ML-methods drastically outperform the lateration method. When one considers set *D* for example, the GP model from Table 8 results in an improvement of the p50 error of 43.5% and an improvement of the p95 error of 44.8% when compared to Table 5. For set *E*, the GP model from Table 8 results in an improvement of the p50 error of 58.2% and an improvement of the p95 error of 56.4%.

#### 3.2.3. Comparison of ML Models and Relative Multilateration

In this section, the performance of the relative RSS multilateration schemes, described in Section 2, is discussed and compared to the classical multilateration implementation and with the ML models. The results for the relative multilateration schemes are summarized in Table 10.

The p50 error of the relative multilateration strategies ranges from 4.10 cm in the case of LED dimming to 13.39 cm in the case of aperture obfuscation, thus resulting in an improved median accuracy compared to the classical lateration method from Table 5. However, with a p95 error exceeding 1 m in some cases, this does not perform as is desired in a robust localization system. If one additionally compares the ML methods to the relative multilateration schemes, one can clearly see that MLP and GP significantly outperform the analytical methods, even at small training set sizes. When considering $r_{rel,max}$ for set *B* for example, the GP model results in an improvement of the p50 of 12% and an improvement of 90% of the p95 error.

When comparing the performance on set *E* of the GP with the relative multilateration approach when using $r_{rel,all}$, one can see that the multilateration algorithm performs slightly better in terms of p50 which is 3.4% lower, but the GP delivers significantly more robust results with an improvement of the p95 error of 55.3%. From these results, it can be concluded that the ML methods clearly outperform the multilateration strategies and are substantially more suited to cope with the environment dynamics scenarios investigated in this work.

## 4. Conclusions

In this work, an experimental evaluation of ML methods for robust RSS-based VLP has been executed. The methods use relative RSS-based input features to provide a more reliable localization system when unknown changes in received signal strength occur due to PD aperture obfuscation or LED dimming. Both ML models deliver accurate localization in the scenario without environmental changes when using absolute intensities with a p50 of 1.97 cm and 1.92 cm for the MLP and GP, respectively. Using relative intensities as features, however, is an added value because it renders independence to the investigated changing system parameters. It was demonstrated that ML methods using relative intensities as features significantly outperform similar multilateration schemes in terms of localization accuracy and robustness in the investigated environmental dynamics scenarios. The scenario where $r_{rel,all}$ is used as input vector is demonstrated to be the most robust. Furthermore, it can be stated that the GP model is better suited than the MLP model because it is more data efficient and provides superior results in terms of p50 and p95 errors on the measured datasets. 

## Figures and Tables

**Table 1:** The system parameters of the experimental set-up.

System Parameters	Parameter Value
Room Dimensions	3 m × 3 m
Height *h*	1.284 m
Number of LEDs	4
LED Lambertian order *m*	1

**Table 2:** Parameters of the VLP receiver in the experimental set-up.

Receiver Parameters	Parameter Value
Photodiode area size	13 mm^2^
TIA Gain	40 k
DC-bias voltage	1.024 V
LPF cut off frequency	36 kHz
Sampling frequency	128 kHz
ADC-range	2.048 V
ADC precision	14 bit

**Table 3:** Summary of the acquired datasets with their reference name and the context describing the experiment circumstances.

Dataset	Size	Context
*A*	177	Set measured with LED current of 350 mA. This dataset is used as training set for the ML methods.
*B*	165	Set measured with LED current of 300 mA.
*C*	127	Set measured with LED current of 250 mA.
*D*	174	Set measured with white chalk dust scattered on the PD surface, LED current 350 mA.
*E*	162	Set measured with sawdust scattered on the PD surface, on the PD surface, LED current 350 mA.

**Table 4:** The p50 and p95 errors expressed in (cm) for the evaluated schemes on dataset *A*.

	Performance on Dataset *A*					
	Input *r*		Input $r_{rel,max}$		Input $r_{rel,all}$	
Configuration	p50	p95	p50	p95	p50	p95
MLP	1.97	6.87	2.34	7.72	2.15	7.55
GP	1.92	6.41	2.03	7.19	2.23	7.17
MLAT	3.28	8.17	4.09	46.06	4.33	41.03

**Table 5:** The p50 and p95 percentiles of the error expressed in (cm) for the classical multilateration algorithm.

Classical Multilateration Model Using the Absolute Intensities *r* as Features		
Data	p50	p95
*B*	10.36	27.30
*C*	25.82	54.86
*D*	16.42	35.38
*E*	32.68	58.47

**Table 6:** The p50 and p95 percentiles of the error expressed in (cm) for the Gaussian process model.

Gaussian Process Model Using $r_{rel,max}$										
	N=50		N=75		N=100		N=125		N=176	
**Data**	**p50**	**p95**	**p50**	**p95**	**p50**	**p95**	**p50**	**p95**	**p50**	**p95**
*B*	5.30	19.45	4.56	15.73	4.14	15.99	3.89	12.38	3.61	11.52
*C*	8.91	20.91	8.61	19.19	8.38	19.32	8.11	18.49	7.77	18.39
*D*	10.55	24.25	10.12	22.46	9.95	21.89	9.83	20.92	9.99	20.53
*E*	14.25	29.47	14.05	26.96	13.95	26.2	14.01	24.03	13.88	22.95

**Table 7:** The p50 and p95 percentiles of the error expressed in (cm) for the Multilayer Perceptron model.

Multi Layer Perceptron Model Using $r_{\mathrm{rel},\max}$										
	N=50		N=75		N=100		N=125		N=176	
**Data**	**p50**	**p95**	**p50**	**p95**	**p50**	**p95**	**p50**	**p95**	**p50**	**p95**
*B*	5.41	39.62	4.31	19.68	4.12	18.69	3.75	15.98	3.59	15.23
*C*	8.77	27.26	8.25	21.78	8.11	20.86	7.69	19.68	7.98	18.72
*D*	10.65	32.65	10.22	22.15	9.88	22.12	9.83	22.12	9.76	20.33
*E*	14.18	55.07	14.00	30.26	13.79	30.95	13.88	27.39	13.93	27.74

**Table 8:** The p50 and p95 percentiles of the error expressed in (cm) for the Gaussian process model for the second relative RSS scheme.

Gaussian Process Model Using $r_{\mathrm{rel},\mathrm{all}}$										
	N=50		N=75		N=100		N=125		N=176	
**Data**	**p50**	**p95**	**p50**	**p95**	**p50**	**p95**	**p50**	**p95**	**p50**	**p95**
*B*	4.00	17.09	3.36	13.77	3.25	12.74	3.20	11.60	3.02	10.82
*C*	8.27	20.44	7.96	19.09	8.03	18.81	7.85	17.59	7.68	18.80
*D*	10.10	20.77	9.89	19.61	9.90	19.67	9.65	19.40	9.27	19.54
*E*	14.02	26.38	14.14	25.27	14.33	25.56	14.11	25.31	13.66	25.52

**Table 9:** 50 and 95 percentile of the error expressed in (cm) for the Multilayer Perceptron model for the second relative RSS scheme.

Multi Layer Perceptron Model Using $r_{\mathrm{rel},\mathrm{all}}$										
	N=50		N=75		N=100		N=125		N=176	
**Data**	**p50**	**p95**	**p50**	**p95**	**p50**	**p95**	**p50**	**p95**	**p50**	**p95**
*B*	5.43	40.57	4.30	30.25	3.70	21.92	3.53	16.43	3.49	15.48
*C*	8.59	45.16	7.88	25.37	7.67	22.29	7.76	19.23	7.65	20.58
*D*	10.80	39.6	9.95	27.13	9.73	21.21	9.68	19.77	9.52	19.81
*E*	14.05	54.54	13.62	40.59	13.99	32.97	13.78	27.93	13.82	27.55

**Table 10:** The p50 and p95 percentiles of the error expressed in (cm) for the relative multilateration schemes.

Multilateration Cross Validation				
	$r_{\mathrm{rel},\max}$ Features		$r_{\mathrm{rel},\mathrm{all}}$ Features	
Data	p50	p95	p50	p95
*B*	4.10	112	4.49	125.67
*C*	6.56	35.77	6.74	47.47
*D*	8.83	62.79	9.16	62.98
*E*	13.39	82.48	13.19	57.15
